# Iron Fortification of Foods for Infants and Children in Low-Income Countries: Effects on the Gut Microbiome, Gut Inflammation, and Diarrhea

**DOI:** 10.3390/nu8080494

**Published:** 2016-08-12

**Authors:** Daniela Paganini, Mary A. Uyoga, Michael B. Zimmermann

**Affiliations:** 1 Laboratory of Human Nutrition, Institute of Food, Nutrition and Health, ETH Zurich, Zurich 8092, Switzerland; daniela.paganini@hest.ethz.ch; 2 College of Health Sciences, Jomo Kenyatta University of Agriculture and Technology, Nairobi 00200, Kenya; muyoga26@gmail.com

**Keywords:** iron fortification, infancy, micronutrient powders, gut microbiome, calprotectin, gut inflammation, diarrhea, enterobacteria, Kenya

## Abstract

Iron deficiency anemia (IDA) is common among infants and children in Sub-Saharan Africa and is a leading contributor to the global burden of disease, as well as a hindrance to national development. In-home iron fortification of complementary foods using micronutrient powders (MNPs) effectively reduces the risk for IDA by ensuring that the iron needs of infants and young children are met without changing their traditional diet. However, the iron dose delivered by MNPs is high, and comparable on a mg iron per kg body weight to the supplemental doses (2 mg/kg) typically given to older children, which increases diarrhea risk. In controlled studies, iron-containing MNPs modestly increase risk for diarrhea in infants; in some cases, the diarrhea is severe and may require hospitalization. Recent in vitro and in vivo studies provide insights into the mechanism of this effect. Provision of iron fortificants to school-age children and iron-containing MNPs to weaning infants decreases the number of beneficial ‘barrier’ commensal gut bacteria (e.g., bifidobacteria), increases the enterobacteria to bifidobacteria ratio and abundances of opportunistic pathogens (e.g., pathogenic *Escherichia coli*), and induces gut inflammation. Thus, although iron-containing MNPs are highly effective in reducing IDA, they may increase gastrointestinal morbidity in infants, and safer formulations are needed.

## 1. Iron Deficiency Anemia in Africa

The World Health Organization (WHO) estimates that 62.3% of preschool children in Africa are anemic [1], and it is thought that about 50% of anemia cases in Africa are due to iron deficiency [2]. Anemia is particularly high in infants during the weaning period (i.e., after six months of age) for several reasons: inadequate iron stores at birth due to low birth weight, prematurity, lack of delayed umbilical cord clamping, and maternal anemia; increased requirements for rapid growth and erythropoiesis due to blood loss from parasitic infections; high infection burden (e.g., malaria) that increases hepcidin and reduces iron absorption and utilization; and insufficient bioavailable iron in complementary foods [3]. In rural Kenya, 71% of six month old infants are anemic, mostly due to iron deficiency [4]. Iron deficiency anemia (IDA) is one of 10 leading global risk factors for disease, disability, and death, much of this occurring in young children [5]. IDA impairs cognitive and motor development in infants and young children [6], and this may be irreversible or only partially reversible by later provision of iron [7,8]. Because IDA during early childhood may reduce IQ, it has not only serious health costs but also economic consequences and it is therefore an obstacle to national development [6,9]. It is estimated that if iron fortification could reach 50% of the population in the WHO African sub region, it would likely avert 570,000 disability adjusted life years (DALYs) annually [10]. In an analysis of 10 developing countries, the median value of annual productivity losses due to IDA was ≈US $0.32 per capita, or 0.57% of the gross domestic product [9]. Infants from the poorest families are the most vulnerable, and it is they who stand to gain the most by its reduction [3]. Thus, IDA is a leading cause of disability and a hindrance to development in Sub-Saharan Africa, and effective and safe interventions are urgently needed.

## 2. Iron-Containing Micronutrient Powders

Provision of sufficient dietary iron during the weaning period of infancy is challenging, because breast milk contains little iron [11], and complementary foods are mainly carbohydrates and/or legumes and typically rich in iron absorption inhibitors (e.g., phytic acid, polyphenols). In-home iron fortification, where the caregiver adds vitamins and minerals to weaning foods at home using micronutrient powders (MNPs) containing iron reduces IDA risk in African infants, even in areas with high prevalence of infection and inflammation [12,13,14,15]. The most widely-used MNP is the “Sprinkles” formulation; it provides a high dose of 12.5 mg iron/day in order to deliver adequate absorbed iron even when added to complementary foods rich in inhibitors of iron absorption. The WHO concluded that home fortification with MNPs is an effective intervention to reduce IDA in children 6 to 23 months of age [16], and can be as effective as iron supplements [17]. Currently, in-home MNP fortification programs are in place or planned in 36 countries worldwide including 10 in Sub-Saharan Africa [18]. However, the safety of high dose iron MNPs is uncertain. A controlled trial among Tanzanian preschool children showed that oral iron supplements (12.5 Fe mg/day, the same dose as in the “Sprinkles” MNP) increased the risk of serious adverse events, hospitalizations, and mortality [19]. A subsequent WHO consultation examining the trial did not recommend the use of high dose iron MNPs in malaria-endemic areas because of concerns about potential increases in infection [20].

A low-iron dose MNP (“MixMe”) has been developed containing 2.5 mg Fe as sodium iron ethylenediaminetetraacetate (NaFeEDTA), as a potentially safer alternative to MNPs that contain 12.5 mg Fe/day [21]. NaFeEDTA is a chelated form of iron that has high bioavailability in inhibitory food matrices, and is a WHO recommended iron fortificant for inhibitory foods [22]. However, the current Acceptable Daily Intake (ADI) for EDTA limits the iron dose that can be provided by NaFeEDTA for infants to only 2–3 mg iron per day. In the “MixMe” MNP, NaFeEDTA is given with ascorbic acid and phytase, two enhancers of iron absorption [23]. This formulation improves iron absorption from inhibitory food matrices like maize porridge [21] but has shown weak efficacy in school-age children in South Africa [13] and preschool children in Kenya [14]. We recently reported that the “MixMe” MNP did not significantly reduce IDA in a one year controlled trial in six month old Kenyan infants [4], likely because the absorbed iron dose was inadequate due to the high prevalence of infections reducing absorption and the low iron dose did not cover the increased iron requirements for rapid growth and erythropoiesis in weaning infants. Thus, it is unclear how low the MNP daily iron dose can be to retain efficacy against anemia but at the same time reduce the adverse impact on the gut by reducing the amount of unabsorbed iron that enters the colon. We recently demonstrated that a dose of 6 mg of iron as ferrous sulfate or NaFeEDTA administered with food to adults did not generate non-transferrin bound iron (NTBI), a potentially toxic form of unbound iron in serum generated by higher iron doses [24].

## 3. Iron Intake and Diarrhea

Increasing iron intake through supplementation or MNPs may increase diarrhea in infants and children. A study of iron-fortified infant formula reported a significantly higher diarrhea incidence in infants receiving iron-enriched milk for six months [25]. In an early systematic review of controlled trials of iron supplementation and fortification, provision of iron predicted an 11% greater risk of diarrhea (*p* = 0.04) [26]. In that review, four food fortification studies reported diarrheal outcomes, where three studies provided iron-fortified infant formula [25,27,28] and one provided an iron-fortified infant food [29]; only one reported a significantly higher incidence of watery diarrhea in infants receiving iron-enriched milk for six months [27]. In a controlled trial in infants given iron supplements from six to nine months, iron increased the risk of diarrhea in those infants with Hb ≥ 110 g/L [30]. Controlled iron supplementation trials (12.5–15 mg Fe/day) in Peru [31] and Bangladesh [32] reported a significant increase in diarrhea. Two very large trials of iron (with folic acid) supplementation in Nepal [33] and Tanzania [19], in which children aged 12 to 35 months received 12.5 mg Fe/day and infants aged 1 to 11 months received 6.25 mg Fe/day, reported cause-specific mortality and diarrhea incidence as secondary outcomes. In Nepal, there was a nonsignificant 21% increase in the risk of death from diarrhea in the iron group (RR 1.21, 95% CI 0.66–2.11), but no difference in diarrhea incidence (RR 0.94, 95% CI 0.84–1.05) [33]. In Tanzania, there was no significant increase in diarrhea incidence with iron (RR 0.92, 95% CI 0.68–1.25) [19]. In a recent systematic review of randomized controlled trials of daily oral iron supplementation (mainly with ferrous iron) in children 4–23 months of age, iron significantly increased vomiting (RR 1.38, 95% CI 1.10–1.73) and fever (1.16, 1.02–1.31), but in the six trials that assessed diarrhea, iron did not increase the risk of diarrhea prevalence (RR 1.03, 95% CI 0.86–1.23) or diarrhea incidence (rate ratio 0.98, 95% CI 0.88–1.09) [34]. In a 45-week intervention study of multiple micronutrient supplementation (containing 18 mg iron as ferrous fumarate) with or without zinc among Tanzanian children (aged 6 to 60 months) with a baseline height-for-age z-score <−1.5 SD, the micronutrient group had more diarrhea (hazard ratio 1.19 95% CI 0.94–1.50), particularly in children with asymptomatic giardiasis at baseline (2.03; 1.24–3.32) [35].

In a double-blind, cluster randomized trial of Ghanaian children aged 6 to 35 months (*n* = 1958) conducted over six months (five months followed by one month of further monitoring), children received a MNP with 12.5 mg Fe/day or a MNP without iron. During the intervention period, there were significantly more hospital admissions with iron (RR 1.23, 95% CI 1.02–1.49) and based on the outpatient register, 83% of the additional cases in the iron group were due to diarrhea; however, this was not significant (RR 1.12, 95% CI 0.86–1.46) [36]. The largest safety study of MNPs to date was a cluster randomized trial in Pakistani infants (*n* = 2746) aged 6 to 18 months who were assigned to a control group, or to receive a MNP containing 12.5 mg iron with or without 10 mg of zinc for 12 months [37]. The incidence risk ratios (IRR) (95% CI) comparing the control group to MNP with and without zinc were: for bloody diarrhea, 1.63 (1.12–2.39) and 1.88 (1.29–2.74) (*p* = 0.003); for severe diarrhea (≥6 stools per day), 1.28 (1.03–1.57) and 1.17 (0.95–1.45) (*p* = 0.07); for admission to hospital with diarrhea, 1.30 (0.71–2.38) and 1.01 (0.54–1.90) (*p* = 0.63). The difference in incidence for bloody diarrhea between the MNP groups and the control group was ≈0.08 per child year which corresponds to one additional episode of bloody diarrhea per year for every 12 to 13 children treated with MNPs. After 18 months of age, when supplementation ceased, in the following six month observation period, there was no difference in bloody diarrhea or severe diarrhea comparing the groups. This is the first large-scale study of MNPs to systematically collect morbidity data during 12 months of supplementation; most previous studies of MNPs had shorter durations, typically two to four months, and did not collect morbidity data through regular household surveillance. In a systematic review on the effectiveness of MNPs in children, Salam et al. (2013) [38] included 17 studies that evaluated the impact of MNP versus no intervention or control. Most of the studies were done on children aged six months to six years of age in developing countries, and most were effectiveness trials evaluating the impact of MNPs in community settings. MNPs were found to be associated with a significant increase in diarrhea (RR 1.04, 95% CI 1.01–1.06) but did not significantly increase the risk of fever. However, it should be emphasized that most studies assessed impacts on nutritional status only and did not rigorously monitor morbidity. Therefore, the available data are equivocal but suggest oral iron supplements and iron-containing MNPs may modestly increase risk for diarrhea in infants. This may be an important risk, as diarrhea contributes to the death of ≈ one in nine under five-year-old children in Sub-Saharan Africa [39].

## 4. Iron and the Gut Microbiome

Because excess body iron can be toxic and there is no active pathway of iron secretion, iron absorption from the diet is carefully regulated. Fractional absorption of iron from fortificants or supplements is low, particularly when given with complementary foods rich in phytic acid, a potent inhibitor of iron absorption. Typically, <20% of iron added to these foods is absorbed [3,40]. In rural African populations with high levels of inflammation and infection, absorption is likely to be even lower, as inflammation increases circulating hepcidin and this further reduces iron absorption [41]. Although increasing dietary iron through fortification or supplementation will deliver more absorbed iron, most of the additional iron passes unabsorbed into the colon. A breast feeding infant receives very little iron in breast milk; concentrations in mature milk are ≈0.47 mg/L [11]. Introduction of an iron containing MNP with 12.5 mg of iron will typically result in more than 10 mg of unabsorbed iron entering the colon each day, a ≈30-fold increase compared to breast milk alone, that provides only about 0.25–0.35 mg Fe/day.

Iron is an essential, growth-limiting nutrient for many gut bacteria, competing for unabsorbed dietary iron [42]. For most enteric gram-negative bacteria (e.g., *Salmonella*, *Shigella* or pathogenic *Escherichia coli*), iron acquisition plays an essential role in virulence and colonization [43,44]. Commensal gut bacteria belonging to the genera *Lactobacillus* and *Bifidobacterium* provide an important ‘barrier effect’ against colonization by pathogens [45], and in contrast to most enteric gram-negative bacteria, lactobacilli do not require iron, but instead rely on manganese [46]. Lactobacilli do not produce siderophores to sequester iron and their growth is similar in media with and without iron [47]. *Bifidobacterium breve*, an important *Bifidobacterium* species in breast-fed infants, can sequester luminal iron using a divalent metal permease [48] but the majority of *Bifidobacterium* species do not produce siderophores or other active iron carriers. An increase in unabsorbed dietary iron through fortification or supplementation may modify the colonic microbiota equilibrium and favor growth of pathogenic strains over these healthy ‘barrier’ strains.

In animal models, iron deprivation increases fecal total anaerobes, *Enterococcus* spp., as well as lactobacilli [49,50]. In rats, we recently reported that iron deficiency increased *Enterobacteriaceae* and *Lactobacillus/Leuconostoc/Pediococcus* spp., but reduced *Bacteroides* spp. and *Roseburia* spp./*Eubacterium rectale*; fecal propionate and butyrate were also significantly decreased [51]. Iron supplementation partially reestablished original gut microbiota composition and led to a full recovery of metabolic activity. Although the rodent gut microbiota differs from that of humans, these studies demonstrate that varying colonic iron leads to major shifts in microbiota abundances in the gut and modifies metabolic activity [51].

The use of in vitro gut fermentation models allows investigation of the gut microbiota without effects of the host and other environmental factors via highly controlled parameters [52]. The in vitro continuous colonic fermentation model using immobilized child gut microbiota can be used to study the impact of dietary changes on the gut microbiota [53,54,55,56,57]. This model provides high cell density, biodiversity, and long-term stability due to the immobilization of the gut microbiota in gel beads reproducing the free cell and sessile bacterial populations in the colon. Using this system, we have studied the impact of iron deficiency and iron supplementation on immobilized healthy Swiss infant fecal microbiota. Fermentation effluent samples were analyzed daily for their microbial composition and metabolites by targeted real-time quantitative PCR (qPCR), 16S rRNA gene 454-pyrosequencing, and HPLC. Low iron conditions decreased *Roseburia* spp./*E. rectale*, *Clostridium* Cluster IV members, and *Bacteroides* spp. while *Lactobacillus* spp. and *Enterobacteriaceae* increased consistently with a decrease of butyrate (−84%) and propionate (−55%) [58]. However, high iron conditions had no discernible impact on the gut microbiota composition and metabolic activity in comparison to normal iron conditions. An important limitation of this model is that it does not take into account host factors, such as the local (i.e., mucosal) or systemic response to varying iron exposure and/or iron-induced changes in the gut microbiota. In agreement with animal studies, these findings suggest habitually low intake of iron may cause a dysbiosis of the gut microbiota and decrease short chain fatty acid production; this may weaken the gut barrier to potential pathogens, emphasizing the importance of the right balance of iron in the diet.

In a controlled trial of iron fortification in Côte d’Ivoire [59], we determined the effect of iron fortification on gut microbiota and inflammation. In this six month, randomized, double-blind, controlled trial, 6 to 14 years old Ivorian children (*n* = 139) received iron-fortified biscuits, which provided 20 mg Fe/day, 4 times/week as electrolytic iron or non-fortified biscuits. At baseline, there were greater numbers of fecal enterobacteria than that of lactobacilli and bifidobacteria. Iron fortification was ineffective; there were no differences in iron status, anemia, or hookworm prevalence at six months. However, there was a significant increase in the number of enterobacteria, a decrease in lactobacilli, and a significant increase in fecal calprotectin, a marker of gut inflammation [60], that correlated with the increase in fecal enterobacteria, in the iron group. This study was the first to show that anemic African children carry an unfavorable ratio of fecal enterobacteria to bifidobacteria and lactobacilli, which is increased by iron fortification. Thus, iron fortification in this population produced a potentially more pathogenic gut microbiota profile and gut inflammation. In contrast, in a recent high dose iron supplementation trial with South African schoolchildren residing in an area with an improved water supply and a lower risk of contaminated food, there were no significant effects on gut inflammation (as measured by fecal calprotectin) or on the gut microbiota [61]. The contrasting data in these two studies suggest that the adverse effects of iron on the gut in children are more pronounced in settings where the hygiene standards are low and the microbiome is likely to be populated by opportunistic pathogens.

## 5. Studies of Iron-Containing MNPs and Infant Gut Microbiota in Africa

Colonization of the human gut begins at birth and depends on many factors, including the mode of delivery (vaginal versus Caesarean), maternal antibiotic use around delivery, hygiene, and prematurity [62]. The relatively simple gut microbiota of breast-fed infants further diversifies during complementary feeding [63]. In a study comparing the gut microbiota among infants from the US, Venezuela, and Malawi, a common pattern was the dominance of bifidobacteria through the first year after birth, where afterwards bifidobacteria numbers fall steadily leading to the establishment of an adult-like gut microbiota at about 3 years of age [64].

We recently performed a controlled intervention using iron-containing MNPs in Kenyan infants consuming home-fortified maize porridge daily for four months [65]. We performed two randomized controlled trials in six month old infants (*n* = 115): in the first, infants received a daily dose of the MNP containing 2.5 mg iron as NaFeEDTA or the MNP without iron; in the second, they received a different MNP containing 12.5 mg iron as ferrous fumarate or the MNP without iron. The primary outcome was gut microbiome composition analyzed by 16S pyrosequencing and qPCR. Secondary outcomes included fecal calprotectin and incidence of diarrhea. We analyzed the trials separately and in combination. In terms of the efficacy of the iron intervention, there was a significant treatment effect of the MNP containing 12.5 mg iron on body iron (*p* = 0.001), serum ferritin (SF) (*p* = 0.004), soluble transferrin receptor (sTfR) (*p* = 0.008), and zinc protoporphyrin (ZPP) (*p* = 0.039). In contrast, there was no significant treatment effect of the MNP containing 2.5 mg iron on any of the iron status indicators. In accordance with previous studies on the human gut microbiota [66,67,68,69], the four dominant phyla in the infants at baseline were Actinobacteria (63%, mainly *Bifidobacteriaceae*), Firmicutes (22%), Bacteroidetes (9%), and Proteobacteria (4%). There was a very high prevalence of potential enteropathogens in the gut of the infants. From all analysed fecal samples during the study, we detected *Bacillus cereus* in 39.5%, *Staphylococcus aureus* in 65.4%, *Clostridium difficile* in 56.5%, members of the *Clostridium perfringens* group in 89.7%, and *Salmonella* in 22.4% of the samples. Furthermore, we detected enteropathogenic *E. coli* (EPEC) in 65.0%, enterotoxigenic *E. coli* producing heat-labile toxin (ETEC LT) in 49.2%, ETEC producing heat-stable toxin (ETEC ST) in 7.0%, enterohaemorrhagic *E. coli* producing shiga-like toxin 1 (EHEC stx1) in 9.6%, and EHEC stx2 in 8.5% of the samples. There was a significant increase in enterobacteria, particularly *Escherichia/Shigella*, the enterobacteria/bifidobacteria ratio, and *Clostridium* spp. in the iron groups compared to the control. There was a significant treatment effect on the sum of the pathogenic *E. coli* at endpoint (*p* = 0.029), with higher concentrations in the iron groups (6.0 ± 0.5 log gene copy number/g feces) versus (4.5 ± 0.5) in the no iron groups. Abundances of closely related species can predict susceptibility to intestinal colonization by pathogenic bacteria [70]. The increase in enterobacteria with iron may have encouraged colonization by potentially pathogenic members of the genus *Escherichia/Shigella* spp., evidenced by the higher abundances of this genus in the iron groups at endpoint, and in particular of pathogenic *E. coli*. Gut inflammation, assessed by fecal calprotectin, was significantly higher in infants receiving iron (229.2 ± 1.9 µg/g) than in the no iron groups (123.3 ± 2.1 µg/g) (*p* = 0.002). There were no significant differences in fecal acetate, propionate, or butyrate concentrations between the iron and no iron groups during the intervention. During the trial, 27.3% of infants in the iron-containing MNP groups required treatment for diarrhea versus 8.3% in the no iron groups. This controlled study demonstrates that provision of iron-containing MNPs to African infants causes an adverse shift in the gut microbiome, increases gut inflammation, and abundances of enteropathogens.

This review focuses on the effects of iron on the gut microbiome, but it should be noted that higher iron intakes may increase the risk of diarrhea and enteropathogen infections through other mechanisms [71]. These include increased permeability of the small intestine with iron supplementation [72,73,74], metal-induced formation of free radicals and increased oxidative stress [75], and/or iron impairment of the immune response to pathogens [76].

There is an urgent need to find safer formulations of iron fortification for African infants. This could be done in several ways. First, it is possible that the iron dose in MNPs could be reduced from the current level of 12.5 mg Fe/day to 5–6 mg Fe/day, while maximizing absorption, in order to reduce the amount of iron entering the colon. This could be done by including components in the MNP sachet that enhance iron absorption, such as iron in the form of NaFeEDTA, ascorbic acid, and/or an exogenous phytase. In children aged 24 to 31 months, comparing 4 mg iron as ferrous sulfate (FeSO_4_) or a mixture of 2 mg each of iron as FeSO_4_ and NaFeEDTA, iron absorption was ≈50% higher from the food fortified with the 1:1 mixture of FeSO_4_/NaFeEDTA than from the food fortified with FeSO_4_ [77]. Secondly, the MNP could include components that mitigate the adverse effects of fortification iron on the gut microbiome, such as a prebiotic, that could maintain/promote growth of the beneficial ‘barrier’ bacteria. Human milk oligosaccharides (HMOs) are natural prebiotics found in rich amounts in human breast milk [78,79,80] that support growth of beneficial commensals, such as bifidobacteria, in the infant gut. During weaning, as breast milk is gradually replaced by complementary foods, intake of HMOs fall; addition of another prebiotic to MNPs, such as galacto-oligosaccharides (GOS) might be beneficial. GOS is a well-established prebiotic with increased selectivity towards *Bifidobacterium* spp. that express a higher activity of β-galactosidase than many other bacteria colonizing the colon. Lactobacilli can also utilize GOS and express high levels of β-galactosidase [81]. Therefore, GOS increases the population of bifidobacteria and lactobalcilli, may increase short chain fatty acid production [82], and decrease gut pH [83], thereby providing a less favorable growth environment for enteric pathogens and enhancing the barrier against their colonization of the infant gut.

In conclusion, more research is needed to better understand the effects of varying nutritional iron on the gut environment and gut microbiota of infants and children from low-income countries, and to improve the safety profile of MNPs. These studies should be prioritized as in-home fortification programs with MNPs are already in place or planned in 36 low- and middle-income countries including 10 in Sub-Saharan Africa [18].

## References

[1] World Health Organization (WHO) (2015). The Global Prevalence of Anaemia in 2011.

[2] Kassebaum N.J., Jasrasaria R., Naghavi M., Wulf S.K., Johns N., Lozano R., Regan M., Weatherall D., Chou D.P., Eisele T.P. (2014). A systematic analysis of global anemia burden from 1990 to 2010. Blood.

[3] Zimmermann M.B., Hurrell R.F. (2007). Nutritional iron deficiency. Lancet (Lond., Engl.).

[4] Barth-Jaeggi T., Moretti D., Kvalsvig J., Holding P.A., Njenga J., Mwangi A., Chhagan M.K., Lacroix C., Zimmermann M.B. (2015). In-home fortification with 2.5 mg iron as NaFeEDTA does not reduce anaemia but increases weight gain: A randomised controlled trial in kenyan infants. Matern. Child Nutr..

[5] World Health Organization (WHO) (2002). The World Health Report 2002.

[6] Lozoff B. (2007). Iron deficiency and child development. Food Nutr. Bull..

[7] Lozoff B., de Andraca I., Castillo M., Smith J.B., Walter T., Pino P. (2003). Behavioral and developmental effects of preventing iron-deficiency anemia in healthy full-term infants. Pediatrics.

[8] Grantham-McGregor S., Ani C. (2001). A review of studies on the effect of iron deficiency on cognitive development in children. J. Nutr..

[9] Horton S., Ross J. (2003). The economics of iron deficiency. Food Policy.

[10] Baltussen R., Knai C., Sharan M. (2004). Iron fortification and iron supplementation are cost-effective interventions to reduce iron deficiency in four subregions of the world. J. Nutr..

[11] Dorea J.G. (2000). Iron and copper in human milk. Nutrition (Burbank, Los Angeles County, Calif.).

[12] Zlotkin S., Arthur P., Antwi K.Y., Yeung G. (2001). Treatment of anemia with microencapsulated ferrous fumarate plus ascorbic acid supplied as sprinkles to complementary (weaning) foods. Am. J. Clin. Nutr..

[13] Troesch B., van Stuijvenberg M.E., Smuts C.M., Kruger H.S., Biebinger R., Hurrell R.F., Baumgartner J., Zimmermann M.B. (2011). A micronutrient powder with low doses of highly absorbable iron and zinc reduces iron and zinc deficiency and improves weight-for-age z-scores in south african children. J. Nutr..

[14] Macharia-Mutie C.W., Moretti D., van den Briel N., Omusundi A.M., Mwangi A.M., Kok F.J., Zimmermann M.B., Brouwer I.D. (2012). Maize porridge enriched with a micronutrient powder containing low-dose iron as nafeedta but not amaranth grain flour reduces anemia and iron deficiency in Kenyan preschool children. J. Nutr..

[15] World Health Organization (WHO) (2011). Guideline: Use of Multiple Micronutirent Powders for Home Fortification of Foods Consumed by Infants and Children 6–23 Months of Age.

[16] De-Regil L.M., Suchdev P.S., Vist G.E., Walleser S., Pena-Rosas J.P. (2013). Home fortification of foods with multiple micronutrient powders for health and nutrition in children under two years of age. Evid.-Based Child Health.

[17] De-Regil L.M., Suchdev P.S., Vist G.E., Walleser S., Pena-Rosas J.P. (2011). Home fortification of foods with multiple micronutrient powders for health and nutrition in children under two years of age. Chochrane Database Syst. Rev..

[18] Jefferds M.E., Irizarry L., Timmer A., Tripp K. (2013). Unicef-CDC global assessment of home fortification interventions 2011: Current status, new directions, and implications for policy and programmatic guidance. Food Nutr. Bull..

[19] Sazawal S., Black R.E., Ramsan M., Chwaya H.M., Stoltzfus R.J., Dutta A., Dhingra U., Kabole I., Deb S., Othman M.K. (2006). Effects of routine prophylactic supplementation with iron and folic acid on admission to hospital and mortality in preschool children in a high malaria transmission setting: Community-based, randomised, placebo-controlled trial. Lancet (Lond., Engl.).

[20] World Health Organization (WHO) (2007). Conclusions and recommendations of the who consultation on prevention and control of iron deficiency in infants and young children in malaria-endemic areas. Food Nutr. Bull..

[21] Troesch B., Egli I., Zeder C., Hurrell R.F., de Pee S., Zimmermann M.B. (2009). Optimization of a phytase-containing micronutrient powder with low amounts of highly bioavailable iron for in-home fortification of complementary foods. Am. J. Clin. Nutr..

[22] World Healh Organization (WHO), Food and Agriculture Organization (FAO), United Nations Children’s Emergency Fund (UNICEF), GAIN, MI, FFI (2009). Recommendations on Wheat and Maize Flour Fortification.

[23] Food and Agriculture Organization (FAO), World Healh Organization (WHO), JECFA (2012). Safety Evaluation of Certain Food Additives.

[24] Troesch B., Egli I., Zeder C., Hurrell R.F., Zimmermann M.B. (2011). Fortification iron as ferrous sulfate plus ascorbic acid is more rapidly absorbed than as sodium iron edta but neither increases serum nontransferrin-bound iron in women. J. Nutr..

[25] Power H.M., Heese H.D., Beatty D.W., Hughes J., Dempster W.S. (1991). Iron fortification of infant milk formula: The effect on iron status and immune function. Ann. Trop. Paediatr..

[26] Gera T., Sachdev H.P. (2002). Effect of iron supplementation on incidence of infectious illness in children: Systematic review. BMJ.

[27] Brunser O., Espinoza J., Araya M., Pacheco I., Cruchet S. (1993). Chronic iron intake and diarrhoeal disease in infants. A field study in a less-developed country. Eur. J. Clin. Nutr..

[28] Singhal A., Morley R., Abbott R., Fairweather-Tait S., Stephenson T., Lucas A. (2000). Clinical safety of iron-fortified formulas. Pediatrics.

[29] Javaid N., Haschke F., Pietschnig B., Schuster E., Huemer C., Shebaz A., Ganesh P., Steffan I., Hurrel R., Secretin M.C. (1991). Interactions between infections, malnutrition and iron nutritional status in Pakistani infants. A longitudinal study. Acta Paediatr. Scand. Suppl..

[30] Dewey K.G., Domellof M., Cohen R.J., Landa Rivera L., Hernell O., Lonnerdal B. (2002). Iron supplementation affects growth and morbidity of breast-fed infants: Results of a randomized trial in Sweden and Honduras. J. Nutr..

[31] Richard S.A., Zavaleta N., Caulfield L.E., Black R.E., Witzig R.S., Shankar A.H. (2006). Zinc and iron supplementation and malaria, diarrhea, and respiratory infections in children in the Peruvian Amazon. Am. J. Trop. Med. Hyg..

[32] Chang S., El Arifeen S., Bari S., Wahed M.A., Rahman K.M., Rahman M.T., Mahmud A.B., Begum N., Zaman K., Baqui A.H. (2010). Supplementing iron and zinc: Double blind, randomized evaluation of separate or combined delivery. Eur. J. Clin. Nutr..

[33] Tielsch J.M., Khatry S.K., Stoltzfus R.J., Katz J., LeClerq S.C., Adhikari R., Mullany L.C., Shresta S., Black R.E. (2006). Effect of routine prophylactic supplementation with iron and folic acid on preschool child mortality in southern Nepal: Community-based, cluster-randomised, placebo-controlled trial. Lancet (Lond., Engl.).

[34] Pasricha S.R., Hayes E., Kalumba K., Biggs B.A. (2013). Effect of daily iron supplementation on health in children aged 4–23 months: A systematic review and meta-analysis of randomised controlled trials. Lancet Glob. Health.

[35] Veenemans J., Schouten L.R., Ottenhof M.J., Mank T.G., Uges D.R., Mbugi E.V., Demir A.Y., Kraaijenhagen R.J., Savelkoul H.F., Verhoef H. (2012). Effect of preventive supplementation with zinc and other micronutrients on non-malarial morbidity in Tanzanian pre-school children: A randomized trial. PLoS ONE.

[36] Zlotkin S., Newton S., Aimone A.M., Azindow I., Amenga-Etego S., Tchum K., Mahama E., Thorpe K.E., Owusu-Agyei S. (2013). Effect of iron fortification on malaria incidence in infants and young children in ghana: A randomized trial. JAMA.

[37] Soofi S., Cousens S., Iqbal S.P., Akhund T., Khan J., Ahmed I., Zaidi A.K., Bhutta Z.A. (2013). Effect of provision of daily zinc and iron with several micronutrients on growth and morbidity among young children in Pakistan: A cluster-randomised trial. Lancet (Lond., Engl.).

[38] Salam R.A., MacPhail C., Das J.K., Bhutta Z.A. (2013). Effectiveness of micronutrient powders (MNP) in women and children. BMC Public Health.

[39] World Heath Organization (WHO) (2013). World Health Statisitcs 2013.

[40] Tondeur M.C., Schauer C.S., Christofides A.L., Asante K.P., Newton S., Serfass R.E., Zlotkin S.H. (2004). Determination of iron absorption from intrinsically labeled microencapsulated ferrous fumarate (sprinkles) in infants with different iron and hematologic status by using a dual-stable-isotope method. Am. J. Clin. Nutr..

[41] Nemeth E., Tuttle M.S., Powelson J., Vaughn M.B., Donovan A., Ward D.M., Ganz T., Kaplan J. (2004). Hepcidin regulates cellular iron efflux by binding to ferroportin and inducing its internalization. Science (New York, NY).

[42] Andrews S.C., Robinson A.K., Rodríguez-Quiñones F. (2003). Bacterial iron homeostasis. FEMS Microbiol. Rev..

[43] Naikare H., Palyada K., Panciera R., Marlow D., Stintzi A. (2006). Major role for feob in *Campylobacter jejuni* ferrous iron acquisition, gut colonization, and intracellular survival. Infect. Immun..

[44] Bullen J., Griffiths E., Rogers H., Ward G. (2000). Sepsis: The critical role of iron. Microbes Infect./Inst. Pasteur.

[45] Anderson R.C., Cookson A.L., McNabb W.C., Kelly W.J., Roy N.C. (2010). *Lactobacillus plantarum* DSM 2648 is a potential probiotic that enhances intestinal barrier function. FEMS Microbiol. Lett..

[46] Weinberg E.D. (1997). The lactobacillus anomaly: Total iron abstinence. Perspect. Biol. Med..

[47] Pandey A., Bringel F., Meyer J. (1994). Iron requirement and search for siderophores in lactic acid bacteria. Appl. Microbiol. Biotechnol..

[48] Bezkorovainy A., Solberg L. (1989). Ferrous iron uptake by *Bifidobacterium breve*. Biol. Trace Elem. Res..

[49] Tompkins G.R., O’Dell N.L., Bryson I.T., Pennington C.B. (2001). The effects of dietary ferric iron and iron deprivation on the bacterial composition of the mouse intestine. Curr. Microbiol..

[50] Werner T., Wagner S.J., Martinez I., Walter J., Chang J.S., Clavel T., Kisling S., Schuemann K., Haller D. (2011). Depletion of luminal iron alters the gut microbiota and prevents crohn’s disease-like ileitis. Gut.

[51] Dostal A., Chassard C., Hilty F.M., Zimmermann M.B., Jaeggi T., Rossi S., Lacroix C. (2012). Iron depletion and repletion with ferrous sulfate or electrolytic iron modifies the composition and metabolic activity of the gut microbiota in rats. J. Nutr..

[52] Payne A.N., Zihler A., Chassard C., Lacroix C. (2012). Advances and perspectives in in vitro human gut fermentation modeling. Trends Biotechnol..

[53] Le Blay G., Chassard C., Baltzer S., Lacroix C. (2010). Set up of a new in vitro model to study dietary fructans fermentation in formula-fed babies. Br. J. Nutr..

[54] Payne A.N., Chassard C., Banz Y., Lacroix C. (2012). The composition and metabolic activity of child gut microbiota demonstrate differential adaptation to varied nutrient loads in an in vitro model of colonic fermentation. FEMS Microbiol. Ecol..

[55] Cinquin C., le Blay G., Fliss I., Lacroix C. (2004). Immobilization of infant fecal microbiota and utilization in an in vitro colonic fermentation model. Microb. Ecol..

[56] Cinquin C., le Blay G., Fliss I., Lacroix C. (2006). New three-stage in vitro model for infant colonic fermentation with immobilized fecal microbiota. FEMS Microbiol. Ecol..

[57] Zihler A., Gagnon M., Chassard C., Hegland A., Stevens M.J., Braegger C.P., Lacroix C. (2010). Unexpected consequences of administering bacteriocinogenic probiotic strains for salmonella populations, revealed by an in vitro colonic model of the child gut. Microbiology (Read., Engl.).

[58] Dostal A., Fehlbaum S., Chassard C., Zimmermann M.B., Lacroix C. (2013). Low iron availability in continuous in vitro colonic fermentations induces strong dysbiosis of the child gut microbial consortium and a decrease in main metabolites. FEMS Microbiol. Ecol..

[59] Zimmermann M.B., Chassard C., Rohner F., N’Goran E K., Nindjin C., Dostal A., Utzinger J., Ghattas H., Lacroix C., Hurrell R.F. (2010). The effects of iron fortification on the gut microbiota in african children: A randomized controlled trial in cote d’ivoire. Am. J. Clin. Nutr..

[60] Konikoff M.R., Denson L.A. (2006). Role of fecal calprotectin as a biomarker of intestinal inflammation in inflammatory bowel disease. Inflamm. Bowel Dis..

[61] Dostal A., Baumgartner J., Riesen N., Chassard C., Smuts C.M., Zimmermann M.B., Lacroix C. (2014). Effects of iron supplementation on dominant bacterial groups in the gut, faecal scfa and gut inflammation: A randomised, placebo-controlled intervention trial in south african children. Br. J. Nutr..

[62] Penders J., Thijs C., Vink C., Stelma F.F., Snijders B., Kummeling I., van den Brandt P.A., Stobberingh E.E. (2006). Factors influencing the composition of the intestinal microbiota in early infancy. Pediatrics.

[63] Vael C., Desager K. (2009). The importance of the development of the intestinal microbiota in infancy. Curr. Opin. Pediatr..

[64] Yatsunenko T., Rey F.E., Manary M.J., Trehan I., Dominguez-Bello M.G., Contreras M., Magris M., Hidalgo G., Baldassano R.N., Anokhin A.P. (2012). Human gut microbiome viewed across age and geography. Nature.

[65] Jaeggi T., Kortman G.A., Moretti D., Chassard C., Holding P., Dostal A., Boekhorst J., Timmerman H.M., Swinkels D.W., Tjalsma H. (2015). Iron fortification adversely affects the gut microbiome, increases pathogen abundance and induces intestinal inflammation in Kenyan infants. Gut.

[66] De Filippo C., Cavalieri D., Di Paola M., Ramazzotti M., Poullet J.B., Massart S., Collini S., Pieraccini G., Lionetti P. (2010). Impact of diet in shaping gut microbiota revealed by a comparative study in children from europe and rural africa. Proc. Natl. Acad. Sci. USA.

[67] Backhed F., Ley R.E., Sonnenburg J.L., Peterson D.A., Gordon J.I. (2005). Host-bacterial mutualism in the human intestine. Science (New York, NY).

[68] Qin J., Li R., Raes J., Arumugam M., Burgdorf K.S., Manichanh C., Nielsen T., Pons N., Levenez F., Yamada T. (2010). A human gut microbial gene catalogue established by metagenomic sequencing. Nature.

[69] Fallani M., Amarri S., Uusijarvi A., Adam R., Khanna S., Aguilera M., Gil A., Vieites J.M., Norin E., Young D. (2011). Determinants of the human infant intestinal microbiota after the introduction of first complementary foods in infant samples from five european centres. Microbiology (Read., Engl.).

[70] Stecher B., Chaffron S., Kappeli R., Hapfelmeier S., Freedrich S., Weber T.C., Kirundi J., Suar M., McCoy K.D., von Mering C. (2010). Like will to like: Abundances of closely related species can predict susceptibility to intestinal colonization by pathogenic and commensal bacteria. PLoS Pathog..

[71] Kortman G.A., Raffatellu M., Swinkels D.W., Tjalsma H. (2014). Nutritional iron turned inside out: Intestinal stress from a gut microbial perspective. FEMS Microbiol. Rev..

[72] Ferruzza S., Scarino M.L., Gambling L., Natella F., Sambuy Y. (2003). Biphasic effect of iron on human intestinal Caco-2 cells: Early effect on tight junction permeability with delayed onset of oxidative cytotoxic damage. Cell. Mol. Biol. (Noisy-le-Grand, Fr.).

[73] Nchito M., Friis H., Michaelsen K.F., Mubila L., Olsen A. (2006). Iron supplementation increases small intestine permeability in primary schoolchildren in Lusaka, Zambia. Trans. R. Soc. Trop. Med. Hyg..

[74] Li Y., Hansen S.L., Borst L.B., Spears J.W., Moeser A.J. (2016). Dietary iron deficiency and oversupplementation increase intestinal permeability, ion transport, and inflammation in pigs. J. Nutr..

[75] Valko M., Morris H., Cronin M.T. (2005). Metals, toxicity and oxidative stress. Curr. Med. Chem..

[76] Foster S.L., Richardson S.H., Failla M.L. (2001). Elevated iron status increases bacterial invasion and survival and alters cytokine/chemokine mrna expression in Caco-2 human intestinal cells. J. Nutr..

[77] Chang S., Huang Z., Ma Y., Piao J., Yang X., Zeder C., Hurrell R.F., Egli I. (2012). Mixture of ferric sodium ethylenediaminetetraacetate (NaFeEDTA) and ferrous sulfate: An effective iron fortificant for complementary foods for young Chinese children. Food Nutr. Bull..

[78] Fanaro S., Boehm G., Garssen J., Knol J., Mosca F., Stahl B., Vigi V. (2005). Galacto-oligosaccharides and long-chain fructo-oligosaccharides as prebiotics in infant formulas: A review. Acta Paediatr. Suppl..

[79] Biesiekierski J.R., Rosella O., Rose R., Liels K., Barrett J.S., Shepherd S.J., Gibson P.R., Muir J.G. (2011). Quantification of fructans, galacto-oligosacharides and other short-chain carbohydrates in processed grains and cereals. J. Hum. Nutr. Diet..

[80] Le Huerou-Luron I., Blat S., Boudry G. (2010). Breast-v. formula-feeding: Impacts on the digestive tract and immediate and long-term health effects. Nutr. Res. Rev..

[81] Watson D., O’Connell Motherway M., Schoterman M.H., van Neerven R.J., Nauta A., van Sinderen D. (2013). Selective carbohydrate utilization by lactobacilli and bifidobacteria. J. Appl. Microbiol..

[82] Moro G., Minoli I., Mosca M., Fanaro S., Jelinek J., Stahl B., Boehm G. (2002). Dosage-related bifidogenic effects of galacto- and fructooligosaccharides in formula-fed term infants. J. Pediatr. Gastroenterol. Nutr..

[83] Petry N., Egli I., Chassard C., Lacroix C., Hurrell R. (2012). Inulin modifies the bifidobacteria population, fecal lactate concentration, and fecal pH but does not influence iron absorption in women with low iron status. Am. J. Clin. Nutr..

